# Barriers to cervical cancer screening faced by immigrant women in Canada: a systematic scoping review

**DOI:** 10.1186/s12905-018-0654-5

**Published:** 2018-10-11

**Authors:** Mahzabin Ferdous, Sonya Lee, Suzanne Goopy, Huiming Yang, Nahid Rumana, Tasnima Abedin, Tanvir C. Turin

**Affiliations:** 1 Department of Family Medicine, Cumming School of Medicine, University of Calgary, Room G012F, Health Sciences Center, 3330 Hospital Drive NW, Calgary, AB T2N 4N1 Canada; 2 Faculty of Nursing, University of Calgary, Calgary, AB Canada; 3 Alberta Health Services, Calgary, AB Canada; 4 Sleep Center, Foothills Medical Center, University of Calgary, Calgary, AB Canada

**Keywords:** Barriers, Cervical cancer, Screening, Canada

## Abstract

**Background:**

The objective of this scoping study is to review the published literature and summarize findings related to barriers experienced by immigrant women in Canada while accessing cervical cancer screening.

**Methods:**

Electronic databases of peer-reviewed articles and grey literature were searched using comprehensive sets of keywords, without restricting the time period or language. Articles were selected based on the following criteria: (a) the study population consisted of Canadian immigrant women and healthcare providers and other stakeholders serving immigrant women, (b) the research focused on the barriers to accessing cervical cancer screening, and (c) the study was conducted in Canada.

**Results:**

Extracted data were grouped and analyzed, resulting in barriers comprised of six themes: economic barriers, cultural barriers, language barriers, healthcare system-related barriers, knowledge-related barriers, and individual-level barriers. Lack of education, low income, preference for a female physician, lack of knowledge, lack of effective communication, and embarrassment were some of the most common barriers mentioned.

**Conclusions:**

Immigrant access to health services, including cervical cancer screening, is a complex issue concerning a wide range of barriers. Our findings offer insights into barriers to cervical cancer screening in immigrant communities in Canada that can be used to assist policymakers, healthcare providers, and researchers enhance the health and well-being of these populations by mitigating barriers and improving screening.

**Electronic supplementary material:**

The online version of this article (10.1186/s12905-018-0654-5) contains supplementary material, which is available to authorized users.

## Background

Cervical cancer is the third most common yet preventable reproductive cancer among Canadian women [1]. It is estimated that 1550 Canadian women will develop cervical cancer in 2017 and of those 400 will die from it [2]. In Canada, the five-year relative survival rate of cervical cancer is 74%, with survival rates increasing to 93% if diagnosis occurs in stage I-A and rates decreasing to 15% with diagnosis in stage IV-B [3].

Secondary prevention through screening allows for cancer detection prior to symptom development. Early cancer detection results in less aggressive treatments, less time spent in recovery, and improved survival rates, thus reducing cancer morbidity and mortality [4]. The greatest impact of screening derives from its detection of precancerous changes that can be treated before they progress to cancer, as most women treated for precancerous conditions of the cervix have an excellent outcome and will not develop cervical cancer [5]. In Canada, cervical cancer screening was initiated in the 1960s [6] and has since contributed to the declining incidence of invasive cervical cancer and cervical cancer mortality rates [7]. Cervical cancer mortality rates decreased by an average of 2.8% per year between 1992 and 2008 following the introduction of widespread cervical cancer screening [8].

Despite the benefits of cervical cancer screening and the availability of provincially covered healthcare in Canada (meaning all Canadian residents have reasonable access to medically necessary hospital and physician services without paying out-of-pocket [9]), immigrant women continue to be underscreened [10, 11]. This is a critical Canadian health concern, as immigrants constitute 20.6% of the total Canadian population and Canada consistently welcomes thousands of new immigrants every year [12]. In this scoping review, we will summarize the literature with respect to cervical cancer screening barriers faced by immigrant women as identified by immigrant populations, healthcare providers, and stakeholders.

## Method

**Fig. 1:**
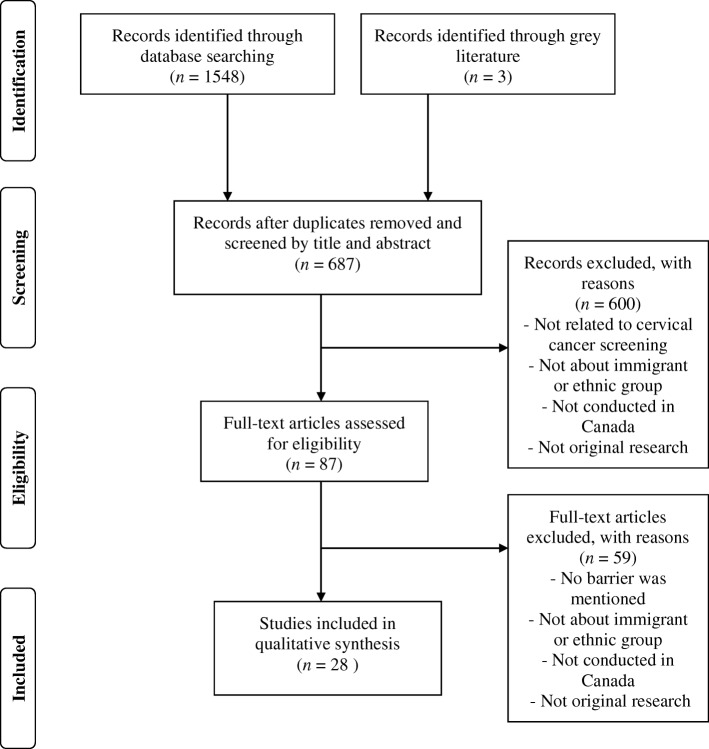
Flowchart for selection of studies

We followed the methodological framework proposed by Arksey and O’Malley to conduct this scoping review [13] and the search process is presented in Fig. 1.

### Data source

Electronic databases of peer-reviewed articles and grey literature were systematically and comprehensively searched. The databases searched are listed in Table 1. A comprehensive set of keywords and Medical Subject Heading (MeSH) terms in four main categories (barriers, cervical cancer, screening, and Canada) were compiled for the search (Table 2). Keywords in each of these areas were joined by the Boolean operator “OR” and all major components were joined by the Boolean operator “AND”.

**Table 1:** Databases searched to identify literature for this review

Published Articles	Grey Literature
MEDLINE	Google
EMBASE	Google Scholar
CINAHL	Proquest (theses and dissertations)
PsycINFO	OpenDOAR (institutional repositories)
Scopus	Health Science Online (HSO)
EMB Reviews (including Cochrane)	Turning Research into Practice (TRIP)
Web of Science	Canadian Institute for Health Information (CIHI)
Family and Society Studies Worldwide	Public Health Agency of Canada (PHAC)
SocINDEX (with full text)	Health Canada
Academic Search Complete	National Institutes of Health (NIH)
Social Work Abstracts

**Table 2:** Search strategy

Keywords for barrier:	
Barrier* [Keyword]; factor* [Keyword]; risk* [Keyword]; risk [MeSH]; “risk factor*” [Keyword]; risk factors [MeSH]; Prejudice [Keyword, MeSH]; self-conscience* [Keyword]; issue* [Keyword]; attitude* [Keyword]; attitude [MeSH]; “Attitude of Health Personnel” [MeSH]; “Attitude to Health” [MeSH]; “Health Knowledge, Attitudes, Practice” [MeSH]; uncertainty [Keyword, MeSH]; mistrust [Keyword]; obstacle* [Keyword]; hurdle* [Keyword]; difficulty [Keyword]; difficulties [Keyword]; obstruction [Keyword]; impediment [Keyword]; Challenge* [Keyword]; confront* [Keyword]; defy [Keyword]; defiance [Keyword]; object* [Keyword]; contest* [Keyword]; question* [Keyword]; “Health Services Accessibility” [Keyword]; hinder*[Keyword]; inhibitor* [Keyword]; roadblock* [Keyword]; block* [Keyword]; pitfall* [Keyword]; “Physician-Patient Relations” [MeSH]; “Communication Barriers” [MeSH]	
Keywords for screening:	
Screening [Keyword]; “Mass Screening” [MeSH]; “preventive test*” [Keyword]; “preventive investigation*” [Keyword]; ‘early diagnosis’ [Keyword]; “Early Diagnosis” [MeSH]	
Keywords for cervical cancer:	
“Cervical cancer” [Keyword]; “Uterine Cervical Neoplasms” [MeSH]; “cervical neoplasm” [Keyword]; “Pap smear” [Keyword]; Papanicolaou Test [MeSH]; “Pap test” [Keyword]; “Vaginal Smears” [MeSH]; “vaginal smear*” [Keyword]	
Keywords for Canada:	
Canada [Keyword]; Canada [MeSH]	

### Study selection

Specific inclusion and exclusion criteria were developed to eliminate the irrelevant articles. The studies were included primarily based on the following criteria:

- The study population consisted of immigrant women and healthcare providers and other stakeholders serving immigrant women
- The study was focused on barriers to cervical cancer screening
- The study was original research
- The study was conducted in Canada

The studies were excluded if:

- They were about refugee or temporary residents or undocumented migrants or aboriginal populations (as healthcare policies and experiences are different and more complicated for these groups than the general Canadian immigrant population [14])
- They were not related to barriers to cervical cancer screening
- They were conference abstracts, editorials, reviews, case reports, consensus statements, guidelines, or described study design only,.

Studies were selected in two stages. First, two independent reviewers (MF, TA) completed title and abstract screening using the inclusion and exclusion. In cases of uncertainty, the study was included for full-text review. Second, full-text review of the selected stage one studies was completed by the same two independent reviewers, and inclusion and exclusion criteria were again applied. Discrepancies between the two reviewers were resolved by discussion between them.

### Data extraction

The following characteristics for each article were recorded and charted in Microsoft Excel: study title, author, year of publication, study objective, study design, data source, location of the study, sample size, study population, participants’ characteristics (e.g., age, country of origin), type of cancer, and barriers discussed.

To develop a description of the research landscape, and to create a thematic construction to present an overview of research that has been conducted in this area, we categorized the included articles based on the barriers identified within those articles. We also further categorized barriers based on patients’, healthcare providers’, and stakeholders’ perspectives. Stakeholders are defined as potential decision makers, program implementers, and program participants from community and health service organizations [15].

## Results

A search of the electronic databases identified 1548 peer-reviewed articles and three grey literature articles. After removing duplicates (*n* = 864), 687 articles underwent full title and abstract screening, resulting in the exclusion of 600 articles (Kappa 0.967). Eighty-seven articles underwent full-text review, resulting in the exclusion of 59 articles (Kappa 0.972). The final number of original research articles for data extraction, analysis, and synthesis was 28. The reference lists of the final 28 articles were also reviewed, and no additional articles met the inclusion criteria. There were no grey literature articles included in the final review.

### Characteristics of selected studies

The characteristics of the selected studies are presented in Table 3. All 28 reported studies were conducted between 1996 and 2016. Most were conducted in the Canadian province of Ontario (*n* = 13), followed by British Columbia (*n* = 6). Study populations were diverse, with a large number of selected studies involving Asian immigrant women. The majority of the studies were on patients’ perspectives on barriers to cervical cancer screening (*n* = 19), three studies discussed healthcare providers’ perspectives on screening barriers, two studies discussed both patients’ and healthcare providers’ perspectives, and one study included stakeholders’ opinions. There were three other studies that used administrative data for their analysis regarding factors associated with underscreening. Most of the studies used qualitative methods (*n* = 12), nine used quantitative methods, and others (*n* = 7) used mixed method (both qualitative and quantitative). Data collection methods and sources included focus groups, surveys, in-depth interviews, administrative databases, and concept mapping.

**Table 3:** Characteristics of selected studies

Study	Year	Study Type	Data Source	Location	Size	Age	Sample
Matuk LC [21]	1996	Qualitative	Focus group questionnaire, telephone interviews, mail surveys, home visits	Windsor, Ontario, Canada	186	Mean 23.6	Newcomer women from the Eastern world, Central and South America, Eastern Europe, and Africa
Woloshin et al. [41]	1997	Quantitative (cross-sectional study)	In-person interview and survey questionnaire	Ontario, Canada	22,448	18–74	Mixed Canadian, Western European, Eastern European, Asian, Native American women
Fitch et al. [32]	1998	Qualitative	Focus group interview	Canada	110	N/A	Sri Lanka, West Indies
Bottorff et al. [33]	2001	Qualitative	Open-ended interview	Canada	35	N/A	Asian, South Asian women
Gupta et al. [18]	2002	Quantitative	Survey questionnaire	Toronto, Ontario, Canada	124	18–60	South Asian women
Jackson et al. [26]	2002	Qualitative	Unstructured interview, focus group interview	Vancouver, British Columbia, Canada, Seattle, Washington, US	87	29–72	Chinese women
Hyman et al. [30]	2002	Qualitative and quantitative	Mail-back questionnaires	Toronto, Ontario, Canada	64	29–71	Physician, 40.6% female
Hyman et al. [29]	2003	Qualitative and quantitative	Mail-back questionnaires	Toronto, Ontario, Canada	112	25–72	Physicians born in Hong Kong, China, Vietnam, and Canada; 25.7% female
Hislop et al. [19]	2003	Qualitative and quantitative (cross-sectional study)	Focus group interview using survey questionnaire	Vancouver, Richmond, British Columbia, Canada	512	20–79	Chinese women
Hislop et al. [20]	2004	Qualitative and quantitative	In-person interviews, focus group, survey questionnaire	British Columbia, Canada	528	20–79	Chinese women
Steven et al. [37]	2004	Qualitative and quantitative	Face-to-face interview	Northwestern Ontario, Canada	105	≥40	Italian, Ukrainians, Finnish, Ojibwa, Oji-Cree women
Donnelly TT [34]	2006	Qualitative	In-depth ethnographic interviews using a semi-structured questionnaire	A western Canadian metropolitan city	15 + 6	49–78	Vietnamese women, 4 male physicians, and 2 female community health nurses
Oelke et al. [31]	2007	Qualitative	In-depth interviews focus groups	Canada	53	N/A	Sikh women
McDonald et al. [11]	2007	Quantitative (cross-sectional study)	Survey (NPHS and CCHS)	New Brunswick, Quebec, Ontario, Manitoba, British Columbia, Saskatchewan, Alberta, Canada	105,062	21–65	White, Black, Hispanic, Arab/West Asian, Chinese, Korean, Japanese, South Asian, Southeast Asian, and Filipino women
Lofters et al. [10]	2007	Quantitative (retrospective cohort)	RPDB and administrative data analysis (billing code)	Toronto, Ontario, Canada	724,584	18–66	N/A
Donnelly TT [28]	2008	Qualitative	In-depth ethnographic interviews using a semi-structured questionnaire	A western Canadian metropolitan city	6	N/A	4 male physicians and 2 female community health nurses
Donnelly et al. [24]	2009	Qualitative	In-depth ethnographic interviews using a semi-structured questionnaire	A western Canadian metropolitan city	15 + 6	N/A	Vietnamese women, 4 male physicians, and 2 female community health nurses
Amankwah et al. [17]	2009	Quantitative (cross-sectional study)	CCHS using questionnaire and interview	All Canadian provinces and territories	76,214	18–65	Chinese, South Asian, Filipino, Other Asian, Black, Latin American, Caucasian, Aboriginals
Woo et al. [38]	2009	Quantitative	Questionnaire	Canada	584	18–60+	Chinese and Euro-Canadian women
Xiong et al. [39]	2010	Quantitative (cross-sectional study)	CCHS	Canada	64,604	18–60+	Asian immigrant (Korean, Filipino, Japanese, Chinese), South Asian (East Indian, Pakistani)
Lofters et al. [23]	2010	Quantitative	Administrative data	Ontario, Canada	2,273,995	25–69	N/A
Lofters et al. [16]	2011	Quantitative (cohort)	Administrative data analysis (billing code)	Ontario’s CMA (Census Metropolitan Area), Canada	455,864	N/A	Women from East Asia and Pacific, Eastern Europe and Central Asia, Latin America and Caribbean, Middle East and North Africa, South Asia, Sub Saharan Africa, USA, Australia and New Zealand, Western Europe
Redwood-Campbell et al. [36]	2011	Qualitative	Focus group interview	Hamilton, Ontario, Canada	77	35–69	Arabs, Chinese, Afghan, Other Asian, Somalian, Spanish women
Black et al. [35]	2011	Qualitative	Focus group interview	New Westminster, Surrey, Burnaby, Coquitlam, Victoria, and Vancouver, British Columbia, Canada	80	20–29	Qualitative
Chang et al. [40]	2013	Qualitative	Focus group interview	Vancouver, British Columbia, Canada	13	Mean 53	Chinese women
Schoueri N [22]	2013	Qualitative and quantitative	Survey (CCHS)	Ontario, Canada	2904	18–69	N/A
Lobb et al. [15]	2013	Qualitative and quantitative	Concept mapping by brainstorming session	Brampton and Mississauga, Ontario, Canada	53	53	Potential decision makers, program implementers, and program participants
Vahabi et al. [27]	2016	Qualitative	Questionnaire, focus group interviews	Ontario, Canada	30	21–69	West Asia (Iran) and South Asia (Pakistan and India)

*CCHS* Canadian Community Health Survey, *NPHS* National Population Health Survey, *RPDB* Registered Persons Database

### Characteristics associated with lower screening rates in immigrant women

The majority of the studies identified that lower level of education was related to low cervical cancer screening rate [10–22]. Specifically, women with less than post-secondary education were less likely to have a Pap test [11, 17, 21]. Education undertaken outside of Canada was significantly correlated with the low prevalence of cervical cancer screening [18]. Lower screening rate was also related to age (e.g., older women [16, 19, 22–24], younger women [16]), and marital status (e.g., single women [11, 17, 19, 22]).

Other factors related to lower rate of cervical cancer screening found from an analysis of administrative data include low income [10, 16], lack of a patient enrolment model in Ontario [16] (a patient enrolment model is a primary healthcare delivery system of patient rostering or patient enrolment, where funding and compensation of participating physicians are tied to the number of patients enrolled [25]), not having a family doctor, not having a female healthcare provider, having a healthcare provider from the same region as the immigrant women [16], and being unable to speak English [10, 16].

### Thematic analysis of barriers to cervical cancer screening faced by immigrant women in Canada

Barriers identified fell into six main categories: (1) economic barriers, (2) healthcare system-related barriers, (3) cultural barriers, (4) language barriers, (5) knowledge-related barriers, and (6) individual-level barriers. Some articles presented findings related to multiple barriers and perspectives, and these were categorized under multiple themes. The categories, while not necessarily mutually exclusive, provide an analytical framework from which to consider immigrant women’s barriers to cervical cancer screening. The barriers described in each article are presented in Additional file 1: Table S1. The themes identified are summarized in Table 4.

**Table 4:** Thematic division of major barriers

Theme	Barriers	N	Reference(s)
Sociodemographic factors associated with under screening	Extreme age groups (older/ younger)	4	[16, 19, 22, 23]
	Lower educational attainment	10	[10, 11, 16–22, 24]
	Less acculturation	2	[18, 19]
	Being single	4	[11, 17, 19, 22]
Economic barriers	Costs (transportation/ child care/ time off work)	5	[15, 24, 26–28]
	Low income/ socioeconomic status	8	[10, 16, 19, 20, 22–24, 28]
	Frequent moving of immigrants	2	[24, 28]
	Intervention not adequately reimbursed	4	[15, 28–30]
Healthcare system-related barriers	No regular doctor	4	[15–17, 22]
	Lack of appropriate reminder system	3	[15, 29, 30]
	Dissatisfaction with patient/provider interaction	3	[15, 21, 32]
	Lack of physician recommendation/consult	9	[15, 20, 22, 27, 31, 33, 37–39]
	Heavy workloads of physicians	1	[34]
	Dearth of acceptable health care provider/ female provider	15	[15, 16, 19, 20, 26, 27, 29–37]
	Long waiting time	3	[15, 27, 32]
	Service is inconvenient/difficult to access	4	[15, 26, 32, 33]
	Lack of appropriate services/referral pattern	1	[15]
	Lack of appropriate educational materials/services	4	[15, 26, 28, 32]
	Difference in the institutionalization of healthcare	1	[40]
Cultural barriers	Cultural differences/religious belief	10	[21, 27–31, 34, 38, 40, 41]
	Stigma	6	[15, 28–31, 40]
	Embarrassment/modesty	11	[15, 20, 26, 30–34, 36–38]
	Preference for traditional/alternative care	1	[40]
	Patriarchy	2	[15, 31]
	Physician-patient hierarchy	2	[28, 34]
Language barriers	Language/communication difficulties	12	[10, 11, 16, 17, 20, 21, 24, 26, 29, 31, 36, 41]
	Lack of interpretation/translation services	2	[26, 28]
Knowledge-related barriers	Lack of information about how to access/navigate services	6	[11, 15, 21, 27, 31, 39]
	Lack of preventive health concept/knowledge of screening tests	9	[15, 18, 21, 27, 31–34, 36]
	Lack of cancer literacy	8	[15, 20, 26–28, 31, 34, 36]
	Belief that screening is not necessary/ ineffective	9	[17, 18, 20, 22, 26, 29–31, 39]
	Physicians unaware of screening guideline	1	[15]
Individual-level barriers	Lack of time	6	[15, 19, 24, 30, 31, 35]
	It is not a priority/self-sacrifice	4	[15, 27, 29, 30]
	Not getting around to it/procrastination	3	[17, 22, 35]
	Concern about cancer diagnosis/ prognosis	4	[15, 20, 26, 32]
	Concern about side effects of treatment	1	[15]
	Fear of procedure (pain/discomfort)	6	[15, 19, 26, 33, 38, 39]
	Sense of confidentiality/privacy	4	[15, 28, 31, 37]
	Limited support/ encouragement	2	[15, 21]

### Economic barriers to cervical cancer screening

#### Patients’ perspective

Low socioeconomic status and low income [19, 20, 22, 24] were considered to be significant barriers to screening. Despite publically funded access to healthcare in Canada, immigrant women found screening tests to be costly due to the incidental expenses and difficulties of transportation and childcare [24, 26, 27]. Many women from low socioeconomic backgrounds earned their income from hourly wages, therefore taking time off work to visit a healthcare provider for screening tests, resulting in the loss of income [24]. Further, some Chinese immigrant women considered screening a waste of government resources [26].

#### Healthcare providers’ perspective

Healthcare providers identified low income, as well as living in a low-income area, as a barrier [28]. As a consequence of low income, immigrant women tended to move frequently, in order to secure more affordable housing. The lack of a permanent address or telephone number created challenges for healthcare providers when attempting to provide reminders for screening [24–28]. Also, hourly wages made it costly for immigrant women to take time off from work to complete screening tests [28]. This financial burden also extended to women who were not actively employed. For example, women who were in the home and taking care of children identified having to arrange for childcare in order to attend appointments as a barrier [28]. Healthcare providers perceived that poverty influences screening, as screening would be considered a lower priority for immigrant populations, who are faced with other more critical challenges and difficulties [24]. In two studies, healthcare providers stated that inadequate reimbursement for the physicians to complete Pap tests contributed to the low rate of screening [29, 30].

#### Stakeholders’ perspective

Stakeholders identified patients’ concerns (the costs associated with the screening test; the costs of, and difficulties associated with, accessing transportation; and the loss of time and wages) as barriers to cervical cancer screening [15]. The lack of financial incentives to support primary care physicians to complete cancer screening was also identified as a barrier to screening [15].

### Healthcare system-related barriers to cervical cancer screening

#### Patients’ perspective

Lack of an acceptable healthcare provider [19, 26, 31–33], specifically a female physician [20, 26, 27, 34, 35], was the most frequent barrier identified in the literature. Most women expressed a preference for a female physician [31, 35–37] irrespective of their ethnicity, as they were more comfortable discussing a Pap test and having an examination with a female physician [26, 33, 37].

Another important factor identified by patients was the lack of physicians’ recommendation for the screening test. [20, 27, 33]. Many participants stated that their family physicians had never informed them of the importance of the Pap test [20, 31, 37, 38]. In some cases, doctors did not think that a Pap test was even necessary [22, 39]. This is important, as many immigrants consider physicians to be trusted and authoritative figures, and if the physician does not recommend a test, the patient will believe it to be unnecessary [27]. Some patients perceived that male physicians were reluctant to refer women for Pap testing to a female physician over concerns of losing their patients to another practitioner [33]. Out of loyalty to their male family physicians, women were hesitant to request a referral to a female physician or a specialized clinic [33] . One study showed that patients believed that physicians educated outside of Canada place less emphasis on disease prevention, and are therefore less likely to discuss screening tests with their patients [27].

Having no regular physician was also identified as a challenge to regular screening tests [17, 22]. Many women were discouraged from asking for and undertaking the screening test because they were dissatisfied with care due to a lack of time spent by physicians explaining, talking, and listening to patients, often caused by the doctor’s heavy workload [34]; a poor patient-doctor relationship [21]; a rushed, non-caring attitude from doctors; and the feeling of being belittled by the conversational tone of the doctors [32].

Immigrant women identified difficulty accessing a screening service (due to the cumbersome system associated with the scheduling of appointments [26, 32, 33], limited office hours [32], long waiting times [27, 32]), as well as challenges in accessing information (due to the paucity of culturally and linguistically appropriate education material [26] and sometimes confusing and contradictory information [32]) as barriers.

Some immigrant women identified differences between the Canadian healthcare system and that of their home country [31.40]. For example, screening is a matter of personal choice in Canada, while it is compulsory and anonymous in China [40], and the healthcare system in India is more focused on curative services, as opposed to the more preventive and health-promoting system in Canada [31]. Preference for the compulsory and anonymous screening services, as well as unfamiliarity with preventive health services in Canada, acted as barriers to screening for these immigrant women.

#### Healthcare providers’ perspective

Similar to patients’ perspectives, healthcare providers identified lack of acceptable female providers as a barrier to screening [29, 30, 34]. Some studies reported that female physicians were consistently more likely to offer to screen compared to male physicians [29]. Inadequate healthcare support for immigrant women and limited access to personnel and funding resources to manage the distribution of healthcare information to immigrants were identified as contributors to low rates of screening [28] .

#### Stakeholders’ perspective

The following barriers were identified in the single study that presented stakeholders’ perspectives [15]: lack of partnerships between public health departments and primary care providers to promote cancer screening; lack of automated reminders to prompt primary care providers to counsel with patients about cancer screening; lack of personal reminders from a credible authority (e.g., Ministry of Health); lack of sufficient numbers of primary care providers, particularly female providers; and, the paucity of test facilities in convenient locations.

Other challenges identified by stakeholders included: the cumbersome process required to access tests, as women had to go through a family physician; lack of time for patients to talk about cancer screening with their primary care provider; delays in appointments (e.g., long wait, inconvenient times); discontinuation of some successful cancer screening programs by the health system; and, finally, a lack of well-translated and culturally appropriate education materials.

### Cultural barriers to cervical cancer screening

#### Patients’ perspective

Cultural differences, social stigma, and beliefs, including religious beliefs, appeared to be major barriers to cervical cancer screening [21, 27, 31, 33, 40, 41]. In many cultures, a woman’s body was considered to be private [21, 31, 34], and there were close links drawn between their sexuality and the Pap test [40]. Many believed that sexual promiscuity was a cause of cervical cancer [40]. Moreover, in some immigrant communities, both sexuality and Pap testing were considered to be too taboo for frank discussion [31, 40]. Many believed that talking, or even thinking, about these issues would result in inviting disease into their lives [27]. Some participants were strongly discouraged by family and community members to discuss such topics [31]. Influenced by these cultural contexts, many women considered it inappropriate to disclose personal information related to their sexual activity and expose their body parts for the screening test [21, 33, 36]. They felt shy, uncomfortable, and embarrassed discussing and/or undertaking a Pap test irrespective of the sex of the physician [20, 26, 31, 33, 34, 36–46]. In some populations, marital status was related to acceptability of screening, as evidenced by expressed reservations in screening unmarried women [33]. Although immigrant women tended to prefer physicians from similar lingo-cultural backgrounds [27], two studies reported that this similarity might prevent the physicians from engaging in discussion around sexual health, including screening [27, 33].

Some cultural barriers were ethnicity specific. For example, Chinese immigrant women preferred Chinese medicine to Western medicine due to its older history, a more holistic approach to health, and allowance for influences on health that may be neither visible nor detectable with available technology. This resulted in a lower rate of cervical cancer screening among Chinese immigrant women [40]. In a study among Vietnamese women, lack of information was identified as a major cause of non-screening, yet many patients also indicated that they could not ask for information from their doctors due to the culturally imposed hierarchical doctor-patient relationship [34]. In Sikh culture, the head of the household usually makes important decisions regarding healthcare. Therefore, female patients were unable to access cancer screening unless approved to do so by the head of the family, or by their partner [31].

Lower levels of acculturation (the process of cultural and psychological change that results following a meeting between cultures [42]) were identified as a significant barrier to accessing and undertaking cervical screening among Chinese and South Asian women [18, 19].

#### Healthcare providers’ perspective

Patients’ discomfort in discussing cervical cancer and their refusal to undergo a Pap test, due to their sociocultural and religious values, were identified by the healthcare providers as barriers to screening [29, 30]. Additionally, one study noted that awareness of immigrant women’s cultural perceptions resulted in physicians feeling uncomfortable in discussing these matters with them [28].

Ensuring patients’ confidentiality was another issue of concern reported by physicians in one study. The possibility of being diagnosed with cervical cancer might imply promiscuity, which in turn might reflect negatively on the individual’s moral character within their cultural context, and possibly lead to judgment from community members. This possibility acts as a deterrent, as the women are intimidated and thus avoid screening [28].

Physicians also found it difficult to communicate with patients due to the hierarchical patient-physician relationship that exists within a patient’s culture [28, 34].

#### Stakeholders’ perspective

Aside from religious belief, modesty, embarrassment, and patriarchy, stakeholders reported that failure to respect or accommodate the cultural and social customs and practices of immigrants contributed to low rates of cervical cancer screening among this group. The lack of ethnic representation among primary care providers and technicians was also identified as a contributing factor [15].

### Language barriers to cervical cancer screening

#### Patients’ perspective

Inability to communicate effectively due to lack of fluency in one of the official languages (English or French) was a significant barrier to screening [11, 17, 20, 21, 24, 26, 31, 36, 41]. This affected immigrant women in accessing healthcare services, scheduling appointments, and discussing and understanding healthcare providers [20, 41]. Lack of interpretation services was also identified as a barrier [26].

#### Healthcare providers’ perspective

Healthcare providers also identified communication as a barrier to screening if the patient did not speak one of the official languages of Canada [29]. They acknowledged the need for better access to interpretation services and translation of educational materials related to cervical cancer screening and noted a lack of funding for this as a major obstacle to this [28].

### Knowledge-related barriers to cervical cancer screening

#### Patients’ perspective

Lack of knowledge was identified as a major barrier to cervical cancer screening among immigrant women for a myriad of reasons. First, some immigrant women reported they could not easily access or navigate the healthcare system, particularly during their initial years in Canada, as they were unaware of the availability and location of health services [11, 21, 27, 31, 39]. Second, many immigrant women reported they had limited knowledge of, and/or misconceptions about, cervical cancer and its risk factors [20, 26, 27, 31, 36]; for example, limited knowledge of the cervix as a part of the body and its location [31]; poor understanding of risk factors resulting in the perception of their being at low risk for disease [34, 36]; and the belief that cervical cancer is caused by an imbalance or bacterial infection, thereby leading to mitigation strategies such as healthy lifestyle and cleanliness instead of screening [34]. Lack of knowledge regarding cervical cancer was related to lower levels of education, language acquisition difficulty, low income, and having a male doctor [20]. Third, a lack of understanding of disease prevention and health promotion was very common among immigrant women [18, 21, 27, 31–34, 36]. Women were not aware of the importance of early detection, specifically in the absence of symptoms [21, 31, 34–36]. They also reported lack of explanation from their healthcare providers about the screening guidelines, the actual test procedure, and implications of test results [18, 21, 27, 31–33, 36]. Lack of knowledge, misconceptions regarding testing, and the perception of being at low risk resulted in many immigrant women concluding that Pap tests were not necessary [17, 18, 20, 22, 26, 31, 39].

#### Healthcare providers’ perspective

Healthcare providers recognized the following challenges to screening: patients’ lack of knowledge about cervical cancer and its screening [28], as well as patients’ refusal to undergo screening based on their personal, cultural, and social, misconceptions that supported the idea that testing was not necessary [20, 29].

#### Stakeholders’ perspective

Stakeholders believed that a lack of physicians’ knowledge contributed to low rates of cervical cancer screening. Primary care providers were perceived to be unaware of screening guidelines and screening programs, and to believe that immigrant women were at lower risk for cervical cancer, resulting in a lack of emphasis on screening [15].

### Individual-level barriers to cervical cancer screening

#### Patients’ perspective

Patient barriers included fears related to the procedure (such as pain, discomfort, and bleeding) [19, 26, 33, 38, 39] and the avoidance of testing due to concerns of receiving a cancer diagnosis, unsuccessful treatment, and a poor prognosis [20, 26, 33]. These were mainly attributed to inadequate explanations and a lack of discussion about cervical cancer screening by healthcare providers. Many women complained that their physicians expected them to discuss only one health problem per visit and book appointments accordingly for any additional problems [27]. This may have contributed to a lack of prioritization of screening issues during appointments. Women frequently reported that they did not have enough time to take the test [19, 24, 31, 35]. Many of these women also had the tendency to place the health and well-being of their families over their own health and well-being [31]. Some women believed that the test was a violation of their confidentiality [31, 37], and others did not take the test due to procrastination [17, 22, 35].

#### Healthcare providers’ perspective

The tendency to prioritize the presenting problem at the time of the visits [29, 30] and lack of both patients’ and physicians’ time during patient visits [30] were acknowledged as challenges to providing screening by the healthcare providers. Patients’ concerns regarding confidentiality were also identified as a barrier to Pap tests [28].

#### Stakeholders’ perspective

Stakeholders recognized the following personal-level factors as challenges for the screening test: fear of emotional or physical discomfort (e.g., pain, invasiveness, embarrassment); fear of the side effects of treatment (e.g., loss of hair, loss of weight, pain); fear of going to the test alone; fear of lack of confidentiality; fear of starting a discussion about cancer or cancer screening with their physicians; fear that cancer will be detected (i.e., stigma, neglect by family); and fear of going to the hospital [15]. They reported that some female patients were not able to access cancer screening unless their partner approved. A brainstorming session with a group of stakeholders also revealed that lack of family and friends who had experienced cancer screening and who could endorse participation, as well as the unimportance attributed to female health in some families, also contributed to the low rate of screening among immigrant women [15].

## Discussion

Based on the findings of 28 research articles, this scoping review offers a summary of the major characteristics and barriers contributing to low rates of cervical cancer screening among immigrant women. We have presented the barriers from the standpoint of the patients, the healthcare providers, and the stakeholders. These cover six different themes: economic barriers, healthcare system-related barriers, cultural barriers, language barriers, knowledge-related barriers, and individual-level barriers. Alignment in the themes between patients’, healthcare providers’, and stakeholders’ perspectives was noted.

It has been reported that women with greater knowledge of cervical cancer risk factors are more likely to receive cervical cancer screening [43]. Based on this review, knowledge-related barriers were shown to be one of the biggest challenges for both patients and healthcare providers, and women’s knowledge of cervical cancer and its screening was significantly affected by other barriers. For example, women with lower levels of education had the least knowledge about cervical cancer [18, 20]. Low socioeconomic status also affected women’s knowledge of screening [24, 28]. Cultural taboos around sexuality and sexual health also discouraged immigrant women from seeking more information concerning cervical cancer and screening [27, 28]. Women who were cared for by female physicians reported being more comfortable discussing this subject, and as a result, had more knowledge compared to women who received healthcare from a male doctor [20, 27]. Immigrant women’s knowledge was also influenced by their linguistic weakness, which significantly affected both communication skills and their ability to understand the available information on cervical cancer screening [41]. Many cultural and religious beliefs (e.g., disease is up to God) [34], alongside a low risk perception of cancer [27, 36], failure to consider screening as priority [31, 34], fear of cancer and its prognosis [15, 33], and fear of the Pap test procedure itself [15] were shown to be significantly associated with a general lack of knowledge about cervical cancer and its detection. To address these beliefs and improve immigrant women’s knowledge, cancer screening-related information needs to be distributed and communicated in a culturally sensitive and linguistically appropriate manner. A proper explanation of the test procedure by physicians can help patients feel more comfortable and can alleviate their fears. Also of interest is that some studies revealed physicians lack knowledge of current screening guidelines [15]. This may be explained in part by the variation in screening guideline recommendations across Canada and worldwide [44].

Influenced by cultural and religious beliefs, many women felt uncomfortable discussing cervical cancer screening, particularly with male physicians [27, 31, 36]. A lack of, or difficulty, accessing female healthcare providers was one of the important barriers to cervical screening. Providing access to female healthcare providers for all immigrant women is challenging, but establishing culturally sensitive screening programs that provide timely access, particularly in immigrant-dense areas, can offer an effective solution.

Despite the religious taboo and social stigma associated with cervical cancer and screening, women from many different cultural backgrounds respected physicians’ opinions and recommendations [27, 36]. Therefore, physicians’ recommendations can play a vital role in increasing cervical screening among immigrant women. Consistent with Canadian studies, US studies demonstrated that physician recommendation for screening contributed to higher participation in cervical cancer screening [45, 46]. Even among Canadian-born women, lack of physician recommendation was associated with patients not having Pap test screening [47]. This suggests that there is a need to ensure that physicians’ knowledge of current screening guidelines is up to date and that there are adequate incentives in place for screening tests to be completed. The presence of an effective reminder system for the primary care team and for patients can also be helpful.

Our analysis showed that recent immigrants were more prone to the risk of never being screened [10, 11, 17, 22, 23]. This was due to low socioeconomic status, language difficulty, and lack of knowledge on how to access and navigate the Canadian healthcare system. During the initial years following their arrival, immigrants were more concerned about their settlement in a new country, and far less concerned with visiting a physician in the absence of symptoms. Furthermore, when considering their own health, they often lacked a preventative health focus, as the healthcare systems of their home countries were more focused on curative health and treatment. In response to this, more innovative ways to familiarize new immigrants with the healthcare system in Canada should be investigated.

Many studies identified that younger, older, and single immigrant women were less likely to participate in screening. Research to further explore these associations is warranted and might reveal exclusive barriers for these groups of women that can be mitigated. There were no studies conducted in Nova Scotia, Prince Edward Island, Newfoundland and Labrador, Nunavut, Yukon, or the Northwest Territories. Ontario and British Columbia (where most of the studies were conducted) are large and populous provinces with large immigrant populations. We expected to have more studies from Quebec, where a large immigrant community is present, but the lack may be explained by our not capturing French language reports. Few studies were undertaken in provinces where unique barriers may be experienced. Further research is suggested to identify cultural, healthcare system, and infrastructural barriers exclusive to these areas. Studies targeting specific cultural and social barriers for defined ethnic groups are also limited and therefore warrants future research.

### Limitations

Although the initial search was not restricted to English-language research articles, we were not able to search strictly non-English databases and websites. This could have resulted in the exclusion of some potential non English-language articles, particularly French language grey literature from Quebec, where French is the official language and which has a fair proportion of immigrants. However, we believe this limitation had minimal impact on the results of the review due to the comprehensiveness of the search.

Populations such as refugees, undocumented, and temporary immigrants were not included in this study; therefore, our ability to extrapolate findings to these groups was limited. Based on our inclusion criteria, only Canadian studies were included. This limits the applicability of findings to other countries, and therefore other healthcare systems. Despite this limitation, since Canadian immigrants are an extremely diverse group, it is likely findings unrelated to the healthcare system will be relevant to other settings.

## Conclusion

Cervical cancer is highly preventable and treatable when detected early through regular screening. Regardless, immigrant women continue to have a low screening rate. Initiatives are warranted to improve immigrant women’s knowledge about cervical cancer screening, as well as how to access services. Ensuring that physicians recommend screening for eligible women is equally important. Immigrant access to health services, including cervical cancer screening, is a complex issue concerning a wide range of barriers. We believe that the barriers summarized in this study can be used to assist policymakers, healthcare providers, and researchers to enhance the health and well-being of these populations by mitigating barriers and improving screening.

## Additional file

Additional file 1: **Table S1.** Barriers to cervical cancer screening reported in the selected studies. (DOCX 39 kb)

## Abbreviations

CCHS: Canadian Community Health Survey
MeSH: Medical Subject Heading
NPHS: National Population Health Survey
RPDB: Registered Persons Database
