# Supplementary material for: Barriers to cervical cancer screening faced by immigrant women in Canada: a systematic scoping review
Source: BMC Womens Health. 2018 Oct 11;18:165. doi: 10.1186/s12905-018-0654-5 (PMC6180489; doi:10.1186/s12905-018-0654-5)
Supplement: Supplementary file 1 — Table S1. Barriers to cervical cancer screening reported in the selected studies. (DOCX 39 kb) [file 12905_2018_654_MOESM1_ESM.docx]

**Additional file 1: Table S1 Barriers to cervical cancer screening reported in the selected studies**

| **Study** | **Barriers** |
| --- | --- |
| **Patient** | |
| Matuk LC [1] | Lower education  Language problems  Not knowing where to find doctors  Poor relationships with doctors  Lack of perception and understanding of the importance of having a cervical smear  Lack of support provided by the medical professionals  Consider disclosing personal information related to their sexual activity and exposing their body parts inappropriate as a result of their cultural background |
| Woloshin *et al.* [2] | Both language and other cultural factors affected the receipt of preventive services by members of the other language group as a barrier to contact with the healthcare system; as a marker for cultural differences about the value of screening; as a communication barrier |
| Fitch *et al.* [3] | Limited access to the doctor  Long waiting time  Non caring attitude of doctors  Information about cancer are confusing and contradictory  Having a Pap test is uncomfortable  Not been aware of how the Pap smear helped in early detection of cervical cancer  Not receiving adequate explanation regarding the procedure |
| Bottorff *et al.* [4] | Unsure about the early detection, particularly in absence of symptoms  Believed that diagnosis would lead to fear, anxiety, and painful or unsuccessful treatment  Reservation about screening unmarried young women  They did not want internal examinations done by male physicians  Feeling uncomfortable and shy discussing Pap testing, particularly with male physicians  Some physicians avoided the topic of Pap testing out of respect for women modesty and comfort  Loyalties to family physicians prevented some women from asking for a referral (e.g., to female physicians or specialized clinics)  Some physicians were reluctant to refer women for Pap testing because of concerns about losing their patients to other practitioners  Women were fearful that the test would involve bleeding, cutting, and pain  Male family physicians  Some women had difficulty accessing the clinic because of cumbersome system for scheduling appointments |
| Gupta *et al.* [5] | Self-perceived lack of need for Pap testing  Lack of knowledge about the test  Fear of discomfort  Lower level of education  Educational training taking place outside of Canada  Lower level of acculturation |
| Jackson *et al.* [6] | **“**Embarrassment  Lack of knowledge  Not necessary  Costly  Cannot communicate or need interpreter  Fear of pain  Fear of finding bad news  Transportation  Inconvenient  Need childcare  Waste of government resources  No good physicians available**”** |
| Hislop *et al.* [7] | Older women  Single women  Less acculturated Chinese women  Lower education  Lower income  Concern about pain with testing  Lack of time for the procedure  Chinese male physician |
| Hislop *et al.* [8] | Lack of knowledge of risk factors for cervical cancer contributed by low education, low income, not fluent in English, having a male doctor  Perceptions that the test is unnecessary  Fear of a cancer diagnosis  Embarrassment with the test procedure  Lack of physician referral |
| Steven *et al.* [9] | Had not been informed about cervical cancer screening  Refusal to have an internal examination  They found it distasteful and resent invasion of their privacy  Found the procedure uncomfortable and embarrassing  Feeling of having their privacy violated  Being shy about having a physician examine them  Preference for a female doctor |
| Oalke *et al.* [10] | Her body (cervix is an unseen and unknown part of body)  Lack of knowledge about cervical cancer screening (minimal knowledge of the Pap test and no ready access to information)  Lack of focus on prevention (seeking healthcare in absence of symptoms is unnecessary and sometimes inappropriate)  Lack of time for self  Patriarchy and respect to family decision  Cervical cancer screening was a topic not to be discussed in the community  Shyness  Unfamiliar with the system  Sex of physician (due to privacy and embarrassment)  Language barrier  Confidentiality  Dearth of acceptable health practitioners  Not been made aware of the Pap test and its importance by their family physicians |
| McDonald *et al.* [11] | Recent immigrants (due to language difficulties, lack of understanding about how the Canadian health system operates or about the services that are available)  Speaking a language at home other than English or French  Less than high school education  Single women |
| Amankwah *et al.* [12] | Never being married  Having less than a post-secondary education  Speaking a language other than English or French  Not having a regular physician  They had not gotten around it  They didn't think Pap test was necessary |
| Woo *et al.* [13] | Embarrassment  Never been told by their doctor  Concern about pain |
| Xiong *et al.* [14] | **“**Have not gotten around to it  Respondent did not think it was necessary  Doctor did not think it was necessary  Did not know where to go / uninformed  Fear  Hate/dislike having one done**”** |
| Redwood-Campbell *et al.* [15] | Knowledge gaps and needs for information on the necessity of cervical cancer screening, how the process is done and the implication of test findings  Misconceptions about cervical cancer causes that could lead to low risk perception and non-participation in screening (e.g., birth control pill, diet, weight)  Preference for female physician  Language barrier  Embarrassment and modesty issue for Chinese and Arabic women |
| Black *et al.* [16] | Difficulty finding a healthcare provider, especially a female provider  Fear and discomfort  Amount of time required  General procrastination |
| Chang *et al.* [17] | Preference of Chinese medicine (it views health more holistically)  Culturally bound perception of sex and sexuality (Chinese women make a close association between Pap testing and sexuality and consider both to be too taboo for frank discussions)  Difference in the institutionalization of healthcare (In China women underwent Pap testing in a compulsory and anonymous manner while it is a matter of personal decision in Canada) |
| Schoueri N [18] | Not getting around to it  Not thinking it was necessary  Doctors not thinking it was necessary  Being 50-69 years old (older)  Being single  Having low education and income  Not having a regular doctor |
| Vahabi *et al*.[19] | Lack of knowledge of the Canadian healthcare system  Difficult accessing female physicians  Language and ethnic match/mismatch in health encounters  Long wait time and enforced prioritization of health concerns  Limited access to transportation and time constraints |
| **Healthcare Provider** | |
| Hyman *et al.* [20] | Patient prefers female doctor  Patient refuses due to modesty  Priority must be given to presenting problem  Patient refuses due to lack of time  Patient refuses thinking test is not effective  Intervention against patient's cultural belief  No reminder system  Not enough time during visit  Intervention not adequately reimbursed  Not comfortable discussing cervical cancer screening |
| Hyman *et al.* [21] | Patient refusal  Priority must be given to presenting problem  Intervention against cultural/religious beliefs  No reminder system  Intervention not adequately reimbursed  Language barriers  Lack of facilities or personnel  Lack of comfort discussing cervical or breast cancer  Pap test not effective  Lack of comfort performing Pap test  Lack of skill performing Pap test |
| Lofters *et al.* [22] | Foreign language  Low income  Low education |
| Donnelly TT [23] | Cultural awareness about the private body makes male physician very uncomfortable with breast and cervical examination  Physicians perceived challenges in communication with Vietnamese women about breast and cervical cancer due to effect of culture  These exams are negatively viewed in the general population  Vietnamese women move often and do not have permanent address and telephone number which make it difficult to notify them about the screening dates  Often the seniors are doing child care so the both parents can be working and they found it costly to the family to take grandma off to get screening when she seems to be just fine  Vietnamese women often get paid hourly and taking several hours to wait for medical check-up at the doctor's office means there will be no pay for these hours  Low socioeconomic status affecting women's knowledge about breast and cervical cancer and it's screening  In Vietnamese culture doctors holding considerable power and women found it difficult to ask their doctor for information  The idea that having a disease could mean disgrace to the family and 'losing face' within the community  Limited manpower and funding resources to distribute healthcare information among the Vietnamese  There is need for more interpreter service and translation of breast and cervical cancer information into Vietnamese but funding is low |
| Lofters *et al.* [24] | Older age  Low income |
| Lofters *et al*. [25] | Being in either the youngest (18-34 years) or the oldest group (50-66 years)  Low income  Lack of educational degree  Not being in a patient enrolment model  Not having a family doctor  Not having a female provider  Having a provider from the same region  Unable to speak English |
| **Both Patient and** **Healthcare Provider** | |
| Donnelly TT [26] | Because of the cultural designation of a women's body as private, the women were uncomfortable and embarrassed with breast and cervical examination  The women did not think they had a health problem unless they were experiencing unusual physical discomfort  Belief that disease and illness are caused by an imbalance within the body  Belief that disease and illness are caused by a bacterial invasion due to either poor personal hygiene or negative environmental conditions  Belief that disease and illness are predetermined by a higher power  Lack of information  Heavy workloads of physicians  Dearth of healthcare providers, especially female healthcare providers  Hierarchical relationship between a women and her physician |
| Donnelly *et al.* [27] | Low socioeconomic status  Inadequate language skills and occupational limitations created by it  They move frequently in order to find housing with cheap rent, thus don't have permanent telephone numbers making it difficult for the physicians to remind them about screening through telephone  Taking time to see a doctor was difficult and costly  Forget about it because they have to struggle with many difficulties in their lives  Poor educational background leading to poor understanding of health issues and practical healthcare behaviors  Limited knowledge |
| **Stakeholder** | |
| Lobb *et al.* [28] | **“Patient’s beliefs, fears, and lack of social support:**  Fear of emotional or physical discomfort about tests (e.g., pain, invasiveness, embarrassment)  Fear of the side effects of treatment (e.g., loss of hair, loss of weight, pain, etc.)  Fear of going to the test alone  Belief about lack of confidentiality  Fear of starting a discussion about cancer or cancer screening with their physician  Fear that cancer will be detected (i.e., stigma, neglect by family)  Fear about going to hospital  Female patient is not able to access cancer screening unless her partner approves  Religious belief about modesty  Lack of family and friends experienced with cancer screening to endorse participation  Females and their health are worthless in some families **Cost:**  Patient is concerned about cost associated with specialized tests  Patient has difficulty accessing transportation, including cost  Patient experiences loss of time and wages to see the primary care provider  **Limited knowledge among residents:**  Limited knowledge about cancer screening tests  Limited accurate knowledge about cancer and risk factors  Limited knowledge about how to access tests  Limited knowledge about the success of cancer treatment  Limited knowledge about the Canadian healthcare system  Limited knowledge about using the health system when not sick  Patient does not prioritize cancer screening  **Ethno-cultural discordance:**  Health system does not respect or accommodate the culture and traditional notions of healthcare among South Asians  Not enough primary care providers and technicians from South Asian cultures or who speak South Asian languages  Education programs do not offer materials that are well translated and culturally appropriate  Not enough female primary care providers  **Limited knowledge among physicians:**  Primary care provider does not emphasize the need for cancer screening  Primary care provider does not equally emphasize the need for mammograms, Pap tests, and fecal occult blood tests  Primary care provider perceives a lower risk of cancer among South Asians  Primary care provider is unaware of guidelines for cancer screening  Primary care provider is unaware of cancer screening programs  Primary care provider lacks regard for patients’ personal choice about whether cancer screening should be completed  Primary care provider does not have financial incentive to ensure cancer screening is completed  **Education programs:**  Do not provide messages through multiple mediums accessed by South Asians (e.g., newspaper, television, etc.)  Do not offer materials that are easy to understand (e.g., use pictures to convey message, low reading level)  Do not offer endorsements from credible sources (e.g., places of worship, schools, South Asian cancer survivors)  Education programs sometimes deliver inconsistent messages  **Health system:**  Not enough partnerships between public health departments and primary care providers to promote cancer screening  The health system does not have automated reminders to prompt primary care providers to talk with patients about cancer screening  The health system does not provide personal reminders from a credible authority (e.g., Ministry of health)  Patient needs to access tests by going through a physician  The region of Peel does not have enough test facilities in convenient locations  Patient has limited time to talk about cancer screening with the primary care provider  Patient experiences delays in getting an appointment (e.g., long wait, inconvenient times)  The health system sometimes discontinues successful cancer screening programs  The region of Peel does not have enough primary care providers**”** |

**References**

1. Matuk LC. Pap smear screening practices in newcomer women. Womens Health Issues. 1996;6:82-8.

2. Woloshin S, Schwartz LM, Katz SJ, Welch HG. Is language a barrier to the use of preventive services? J Gen Intern Med*.* 1997;12:472-7.

3. Fitch MI, Greenberg M, Cava M, Spaner D, Taylor K. Exploring the barriers to cervical screening in an urban Canadian setting. Cancer Nurs*.* 1998;21:441-9.

4. Bottorff JL, Balneaves LG, Sent L, Grewal S, Browne AJ. Cervical cancer screening in ethnocultural groups: case studies in women-centered care. Women Health. 2001;33:29-46.

5. Gupta A, Kumar A, Stewart DE. Cervical cancer screening among South Asian women in Canada: the role of education and acculturation. Health Care Women Int. 2002;23:123-34.

6. Jackson JC, Do H, Chitnarong K, Tu, SP, Marchand A, Hoslop G, Taylor V. Development of cervical cancer control interventions for Chinese immigrants. J Immigr Health*.* 2002;4:147–57.

7. Hislop TG, Deschamps M, Teh C, Jackson C, Tu SP, Yasui Y, Schwartz SM, Kuniyuki A, Taylor V. Facilitators and barriers to cervical cancer screening among Chinese Canadian women. Can J Public Health*.* 2003;94:68-73.

8. Hislop TG, Teh C, Lai A, Ralston JD, Shu J, Taylor VM. Pap Screening and knowledge of risk factors for cervical cancer in Chinese women in British Columbia, Canada. Ethnic Health*.* 2004;9:267-81.

9. Steven D, Fitch M, Dhaliwal H, Kirk-Gardner R, Sevean P, Jamieson J, Woodbeck H. Knowledge, attitudes, beliefs, and practices regarding breast and cervical cancer screening in selected ethnocultural groups in Northwestern Ontario. Oncol Nurs Forum*.* 2004;31:305-11.

10. Oelke ND, Vollman AR. "Inside and Outside": Sikh women's perspectives on cervical cancer screening. Can J Nurs Res. 2007;39:174-89.

11. McDonald JT, Kennedy S. Cervical cancer screening by immigrant and minority women in Canada. J Immigr Minor Health. 2007;9:323-34.

12. Amankwah E, Ngwakongnwi E, Quan H. Why many visible minority women in Canada do not participate in cervical cancer screening. Ethnic Health. 2009;14:337-49.

13. Woo JST, Brotto LA, Gorzalka BB. The role of sexuality in cervical cancer screening among Chinese women. Health Psychol*.* 2009;28:598-604.

14. Xiong H, Murphy M, Mathews M, Gadag V, Wang PP. Cervical cancer screening among Asian Canadian immigrant and nonimmigrant women. Am J Health Behav*.* 2010;34:131-43.

15. Redwood-Campbell L, Fowler N, Laryea S, Howard M, Kaczorowski J. 'Before you teach me, I cannot know': immigrant women's barriers and enablers with regard to cervical cancer screening among different ethnolinguistic groups in Canada. Can J Public Health*.* 2011;102:230-4.

16. Black AT, McCulloch A, Martin RE, Kan L. Young women and cervical cancer screening: what barriers persist? Can J Nurs Res*.* 2011;43:8-21.

17. Chang SC, Woo JS, Yau V, Gorzalka BB, Brotto LA. Cervical cancer screening and chinese women: insights from focus groups. Front Psychol. 2013;4:48.

18. Schoueri N. Cervical cancer screening among immigrant women in Ontario: the influence of acculturation. University of Waterloo; 2013. https://uwspace.uwaterloo.ca/bitstream/handle/10012/7209/Schoueri_Nour.pdf?sequence=1&isAllowed=y.

19. Vahabi M, Lofters A. Muslim immigrant women's views on cervical cancer screening and HPV self-sampling in Ontario, Canada. BMC Public Health*.* 2016;16:868.

20. Hyman I, Singh PM, Meana M, George U, Wells LM, Stewart DE. Physician-related determinants of cervical cancer screening among Caribbean women in Toronto. Ethnic Dis. 2002;12:268-75.

21. Hyman I, Cameron JI, Singh PM, Stewart DE. Physicians and pap testing in the Chinese and Vietnamese communities in Toronto. J Health Care Poor Underserved. 2003;14(4):489-502.

22. Lofters A, Glazier RH, Agha MM, Creatore MI, Moineddin R. Inadequacy of cervical cancer screening among urban recent immigrants: a population-based study of physician and laboratory claims in Toronto, Canada. Prev Med. 2007;44:536-42.

23. Donnelly TT. Challenges in providing breast and cervical cancer screening services to Vietnamese Canadian women: the healthcare providers' perspective. Nurs Inquiry*.* 2008;15:158-68.

24. Lofters AK, Moineddin R, Hwang SW, Glazier RH. Low rates of cervical cancer screening among urban immigrants: a population-based study in Ontario, Canada. Med Care*.* 2010;48:611-8.

25. Lofters AK, Moineddin R, Hwang SW, Glazier RH. Predictors of low cervical cancer screening among immigrant women in Ontario, Canada. BMC Women's Health. 2011;11:20.

26. Donnelly TT. The health-care practices of Vietnamese-Canadian women: cultural influences on breast and cervical cancer screening. C J Nurs Res*.* 2006;38):82-101.

27. Donnelly TT, McKellin W, Hislop G, Long B. Socioeconomic influences on Vietnamese-Canadian women's breast and cervical cancer prevention practices: a social determinant's perspective. Soc Work Public Health. 2009;24:454-76.

28. Lobb R, Pinto AD, Lofters A. Using concept mapping in the knowledge-to-action process to compare stakeholder opinions on barriers to use of cancer screening among South Asians. Implement Sci. 2013;8:37.
